# A Statistical Skull Geometry Model for Children 0-3 Years Old

**DOI:** 10.1371/journal.pone.0127322

**Published:** 2015-05-18

**Authors:** Zhigang Li, Byoung-Keon Park, Weiguo Liu, Jinhuan Zhang, Matthew P. Reed, Jonathan D. Rupp, Carrie N. Hoff, Jingwen Hu

**Affiliations:** 1 School of Mechanical, Electronic and Control Engineering, Beijing Jiaotong University, Beijing, China; 2 University of Michigan Transportation Research Institute, Ann Arbor, Michigan, United States of America; 3 State Key Laboratory of Automotive Safety and Energy, Tsinghua University, Beijing, China; 4 Zhejiang Key Laboratory of Automobile Safety Technology, Hangzhou, China; 5 Center for Ergonomics, Industrial and Operations Engineering, University of Michigan, Ann Arbor, Michigan, United States of America; 6 Department of Biomedical Engineering, University of Michigan, Ann Arbor, Michigan, United States of America; 7 University of Michigan Medical School, Department of Emergency Medicine, Ann Arbor, Michigan, United States of America; 8 University of Michigan Medical School, Department of Radiology, Ann Arbor, Michigan, United States of America; 9 Department of Mechanical Engineering, University of Michigan, Ann Arbor, Michigan, United States of America; Medical University of South Carolina, UNITED STATES

## Abstract

Head injury is the leading cause of fatality and long-term disability for children. Pediatric heads change rapidly in both size and shape during growth, especially for children under 3 years old (YO). To accurately assess the head injury risks for children, it is necessary to understand the geometry of the pediatric head and how morphologic features influence injury causation within the 0–3 YO population. In this study, head CT scans from fifty-six 0–3 YO children were used to develop a statistical model of pediatric skull geometry. Geometric features important for injury prediction, including skull size and shape, skull thickness and suture width, along with their variations among the sample population, were quantified through a series of image and statistical analyses. The size and shape of the pediatric skull change significantly with age and head circumference. The skull thickness and suture width vary with age, head circumference and location, which will have important effects on skull stiffness and injury prediction. The statistical geometry model developed in this study can provide a geometrical basis for future development of child anthropomorphic test devices and pediatric head finite element models.

## Introduction

The head is one of the most frequently injured body regions for children [1] and head injury is the leading cause of pediatric fatality and disability in the United States [2–4]. The geometric variation of the suture, fontanel ossification, and continual bone development with child growth translate into significant differences in skull stiffness across infants and young children of different ages. As a result, a comprehensive understanding of the anatomy and geometry of a pediatric head is necessary to conduct an accurate prediction of pediatric head injuries using injury assessment tools.

The head of an infant or a young child is proportionately larger than that of an adult [5]. The growth rate of a pediatric head generally slows with an increase in age and the growth leads to different inertial properties and head contours at different ages [6]. Head shape also differs between infants and adults, such that the infant skull has greater frontal and parietal prominences, making the face tuck below the brain cage. The fibrous tissues in sutures and fontanels allow the cranium to adapt to the fast growth of the brain [7] and they gradually calcify and close until 2–3 years of age [8]. The composition of a pediatric skull also differs from the adult. At birth, the cranium is a layer of thin plate. With child growth, the cranium becomes a three-layered sandwich structure [9, 10].

Child anthropomorphic test devices (ATDs) and pediatric finite element (FE) models are two major tools to assess child injuries. Child ATDs have been previously utilized to assess child injuries. However, due to the limited available biomechanical data, child ATD head response targets have been generated by scaling the response of the adult midsize male ATDs [11, 12]. This approach assumes that adults and children are geometrically similar and therefore does not account for the effects of structures that are not present in adults. More recently, pediatric FE models have been developed to better understand pediatric head injuries [13–19]. These models have been used to simulate head dynamic responses under impact loading conditions and have provided encouraging results for investigating pediatric head injuries. However, the head geometries for all the above pediatric head FE models are based on single subject of a given age, which could not consider the effects of geometric variability on pediatric head impact response and injury prediction. A parametric pediatric head FE model that can represent the variability in head geometry is necessary, but such a model is not currently available.

The rapid advancement of medical imaging technologies has provided the opportunity to study three-dimensional skull geometry that may be used to develop statistical models of pediatric heads. Neubauer et al [20] described the shape changes during human postnatal ontogeny with morphometric methods using CT scans of 108 dried human crania from newborn to adults. In a previous study, Li et al [21, 22] has developed a parametric pediatric head FE model based on head CT scans from eleven 0–3 month-old (MO) children. As an extension of the previous study, the objective of the current study is to develop a statistical head model for 0–3 year-old (YO) children, which allows to predict the skull geometry including realistic skull size/shape, suture geometry and skull thickness from a given set of parameters such as age and head circumference.

## Methods

### Head CT Image Processing and Landmark Identification

**Fig 1:**
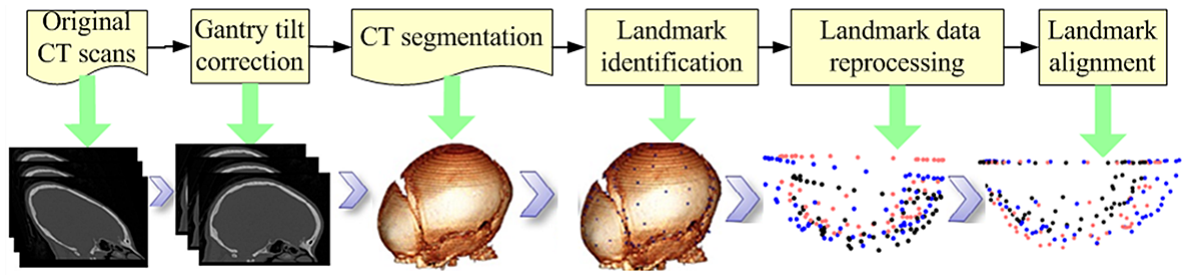
Procedure of CT data processing.

Head CT scans from 56 0–3 YO children without apparent trauma or pathologies were obtained from the University of Michigan Hospital System. The protocol for collecting existing pediatric head CT data was approved by an institutional review board at the University of Michigan (HUM00068381). No consent was given, because the CT data were analyzed anonymously. The CT studies were composed of a set of 512x512-pixel images with an in-plane resolution ranging from 0.273 to 0.410 mm with a slice thickness of 1.25 or 2.5 mm. All images were processed by OsiriX software (Pixmeo, Switzerland) following a procedure shown in Fig 1.

**Fig 2:**
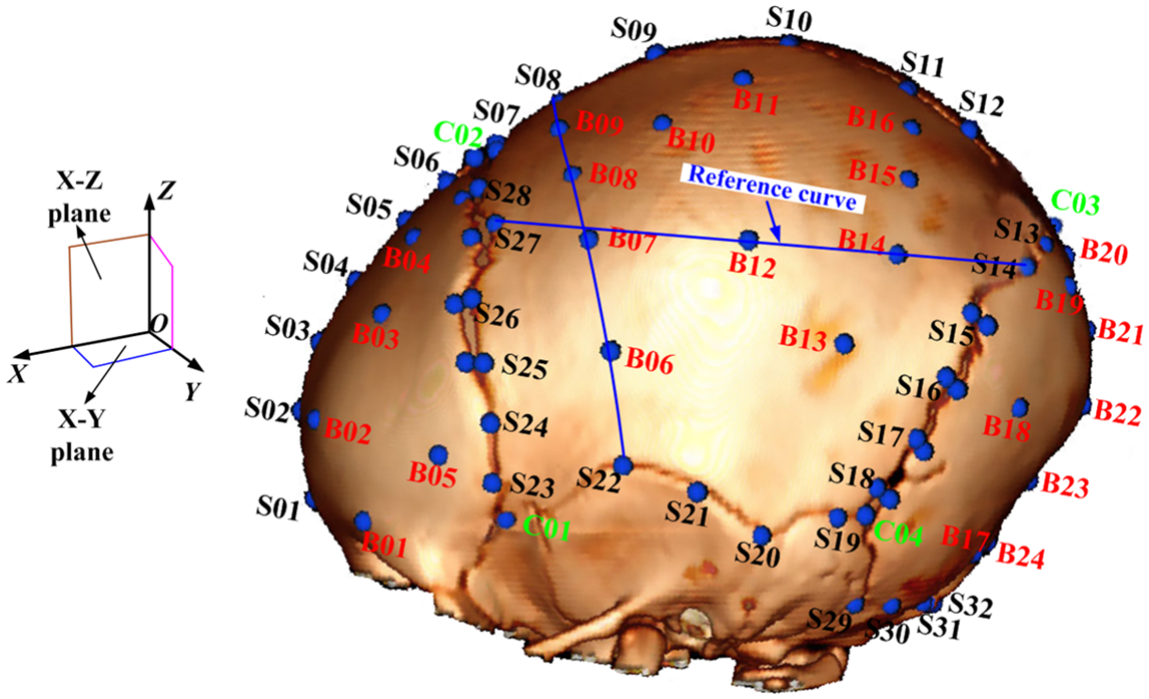
Landmarks on the skull surfaces and suture-bone boundaries. Note: B01 to B24 represent landmarks located at the skull (bone) surface, S01 to S32 represent landmarks located at the suture center lines, and C01 to C04 represent landmarks located at the intersections of the sutures. Landmarks on the skull surface are generally evenly distributed on the reference curves. For example, landmark No. B12 on the skull surface is midway between landmarks No. S27 and S14; and landmark No. B14 is in the middle of landmarks No. B12 and S14.

Several steps are involved in CT image processing as follows:

1. Correct for gantry tilt of the CT scans: Pediatric head geometry may be distorted when the gantry of the CT scanner is tilted in performing the scans, thus gantry tilt correction was first conducted for all the CT scans.
2. Segment the skull from CT data: CT segmentation was conducted for each subject using a window level value of 304 Hounsfield Unit (HU) in OsiriX. Volume rending techniques were used to generate 3D reconstruction of the skull, which has shown to have enough quality to determine the 3D morphologies [23].
3. Identify landmarks and measure skull thickness: The pediatric head can be assumed to be symmetric [14, 24], therefore, the landmarks were identified manually on half of the skull for each subject. Two types of landmarks, anatomic and non-anatomic landmarks, were identified along the suture boundaries and on the skull surface. Most landmarks along the suture are related to anatomical features, which can be identified with relatively high accuracy by a person who is familiar with the pediatric head anatomy. On the other hand, most landmarks on the skull surface generally are not associated with anatomical features, which have to be located based on the relative positions to the anatomic landmarks. To determine the non-anatomic landmarks on the skull surface, a smooth curve along the outside of the skull surface with the shortest distance between a pair of anatomic landmarks was used as the reference curve as shown in Fig 2, which can be identified in OsiriX software. The landmarks on the skull surface were evenly distributed on the reference curves. Because sutures gradually close as a child grows, two landmarks on both sides of the suture across its width were identified when the suture exists. However, if the suture is completely ossified, a single landmark was identified at the centerline of the closed suture. Therefore, fewer landmarks were identified on skulls from older children. For a newborn head, 92 landmarks were identified on half of the head, with 64 landmarks distributed on the suture-bone boundaries, 24 on the skull surface, and 4 at the suture convergences (Fig 2). When all the sutures closed, the 64 landmarks on the suture boundaries were reduced to 32, resulting in a total of 60 landmarks for each subject. Skull thickness values at the landmark locations were manually measured in the reconstructed 3D skull geometry in a direction perpendicular to the tangent plane of skull surface at the landmark location.
4. Reprocess landmark data: As mentioned earlier, the number of landmarks varies with subject age as sutures close. However, to obtain a homologous set of landmarks for statistical analysis, the landmark data were processed such that each pair of landmarks across the suture width was described by a middle landmark (i.e. landmark number of S26, S27, S28 etc) and a suture width. If the suture is closed, zero was assigned for the suture width. Note that we assigned zero for suture width smaller than 0.5 mm in considering the minimum elemental size for further usage of the model in finite element analyses. As a result, the 32 landmarks in the middle of the suture lines and the suture width information corresponding to those landmarks were generated for each subject. Along with the 24 landmarks on the skull surfaces and 4 landmarks at the suture conjunctions, there are a total of 60 landmarks in each pediatric head after data processing.
5. Register and align landmarks: A rigid registration was performed to align the landmark data. Because the infraorbital region was not included in the CT scans of most subjects, the Frankfort plane cannot be used to define the anatomical coordinate system. Instead, the anatomic landmark (S01) located at the lowest position of the mid-sagittal suture near the frontal cranium (Fig 2) was selected as the reference (zero) point. The mid-sagittal plane (the middle position of the sagittal suture) for each head was defined as the X-Z plane. The plane crossing through landmarks S01 and C01 and orthogonal to the X-Z plane was defined as the X-Y plane. To align the landmarks from different subjects, the data for each head was translated to the origin and rotated to align the X-Z plane and X-Y plane.

### Statistical Head Geometry Model Generation

The statistical head geometry model was developed through two steps, including 1) principal component analysis (PCA) and 2) multivariate regression analysis.

Aligned landmarks of 56 subjects were first analyzed using the PCA with suture widths and skull thickness data based on the method presented by Reed and Parkinson [25]. PCA is a widely used tool to express the data on an orthogonal basis that can be more readily analyzed, and achieve data compression. Sixty 3D landmarks of each subject were analyzed for developing PCA. In this study, the spatial coordinates, skull thicknesses, and suture widths at the landmark locations on each subject formed three vectors with length of 60×3 = 180, 25, and 32, respectively. The geometry vectors were appended to form the three geometry matrices with dimensions of 56×180, 56×25, and 56×32 for landmark coordinates, thicknesses, and suture widths. These matrices were then analyzed using PCA, and the first few PC scores covering 95% of geometric variances were stored for further usages.

**Fig 3:**
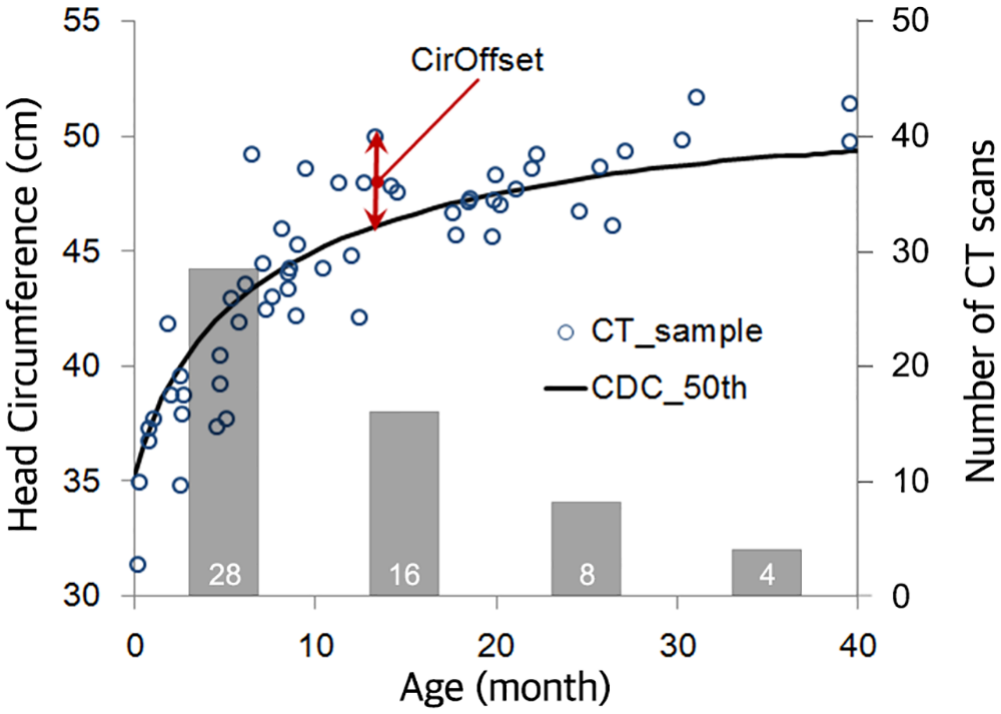
Pediatric head circumference and CirOffset relative to the CDC median head circumference curve.

To associate principal component (PC) scores from the PCA representing head geometry with a given set of predictors, such as age and head circumference, a multivariate regression model was developed based on the procedure presented previously [21, 22, 25]. This statistical model can be used to further develop parametric pediatric head FE models and investigate pediatric head injuries under impact conditions. As one of the predictors for the head geometry model, the head circumferences were measured using the closed polygon option in the 3D mode in OsiriX. A high correlation (R = 0.896) between age and head circumference was observed for the current dataset, which would affect the accuracy of the regression coefficients if they were used directly in the regression model. To eliminate the correlation, a parameter *CirOffset* was introduced to represent the difference between the head circumference from a subject and the 50^th^ percentile head circumference at the subject’s age provided by the CDC growth chart [26]. Fig 3 illustrates the difference between each subject’s head circumference and the 50^th^ percentile growth chart predictions. In this study, the head circumferences measured for the 56 pediatric subjects reasonably correlated (R = 0.902) with those reported from the CDC growth chart as shown in Fig 3, indicating that the current sample can represent the 0–3 YO population well. In order to use predictors to predict subject-specific PC scores, in turn, to predict detailed head geometric features (head size/shape, skull thickness, and suture width), a regression model was generated as follows,

$$S_{K}^{T}\text{=}CF\text{+}\varepsilon^{\text{T}}$$

where ***F*** is the feature matrix, ***C*** is the coefficient matrix, ***ε*** ^T^ is a vector of zero-mean and normally distributed residuals. ***C*** was obtained using standard least-squares techniques. The ***F*** in this study is age and *CirOffset*.

As the final step, predicted landmark points, skull thickness and suture width can be constructed by inputting the corresponding $S_{iK}^{T}$ into the equation (4), and then the final skull geometry can be modeled by fitting a template model based on the predicted landmarks and the skull thickness and suture widths.

### Linear Mixed Model

A linear mixed model was built using SAS (SAS Institute Inc., Cary, NC, USA) to test whether age, head circumference, and landmark location are significant predictors for suture width and skull thickness. A stepwise procedure was used to find the significant predictors starting with main effects from single parameters and adding interaction terms between the significant predictors in the following steps. The model with the lowest Akaike Information Criterion (AIC) score was selected.

## Results

### Landmark Prediction

**Fig 4:**
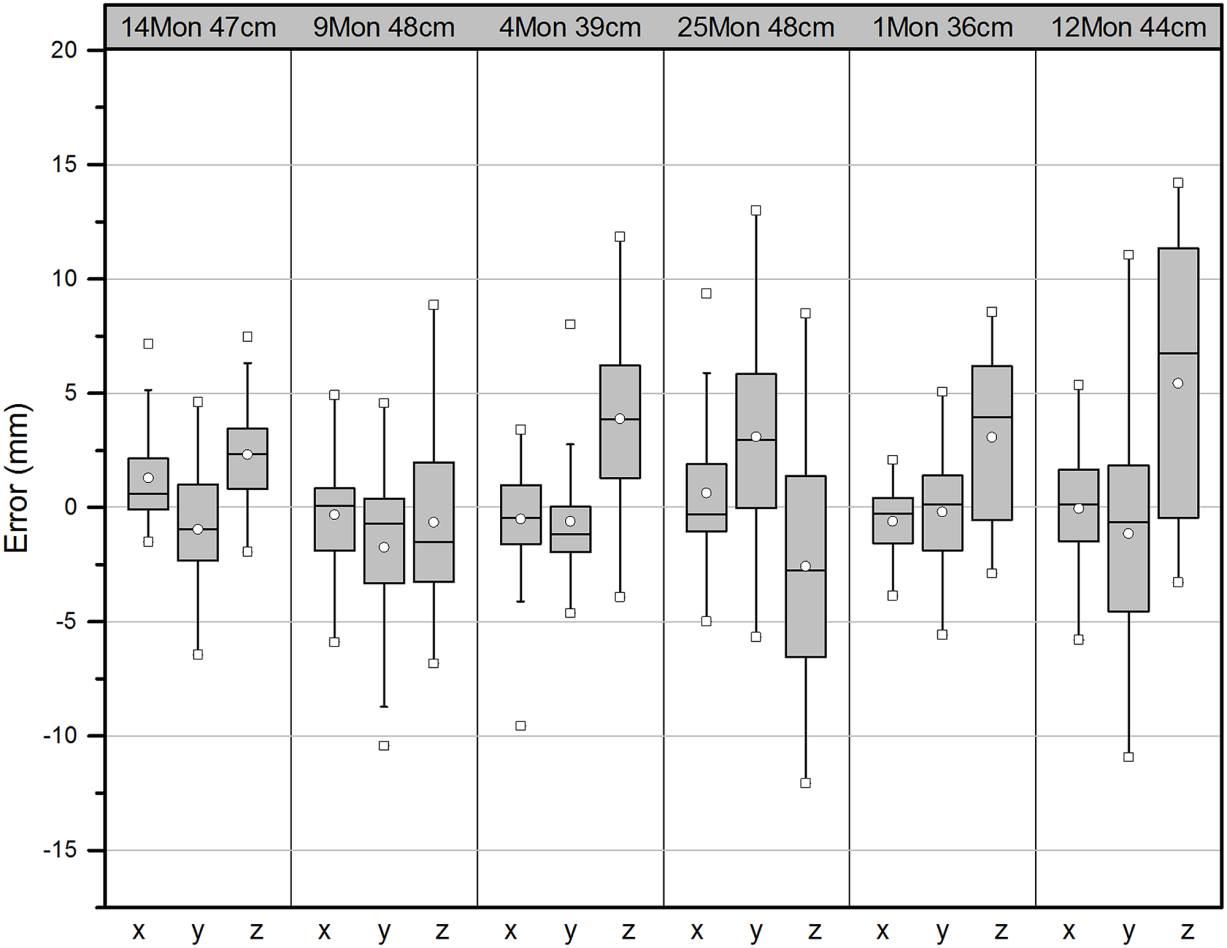
Box plots of signed error in mm between predicted landmark coordinates and the actual landmarks collected from CT data of sampled six subjects. Top row represents the age in month and the head circumference in cm of the subjects.

**Fig 5:**
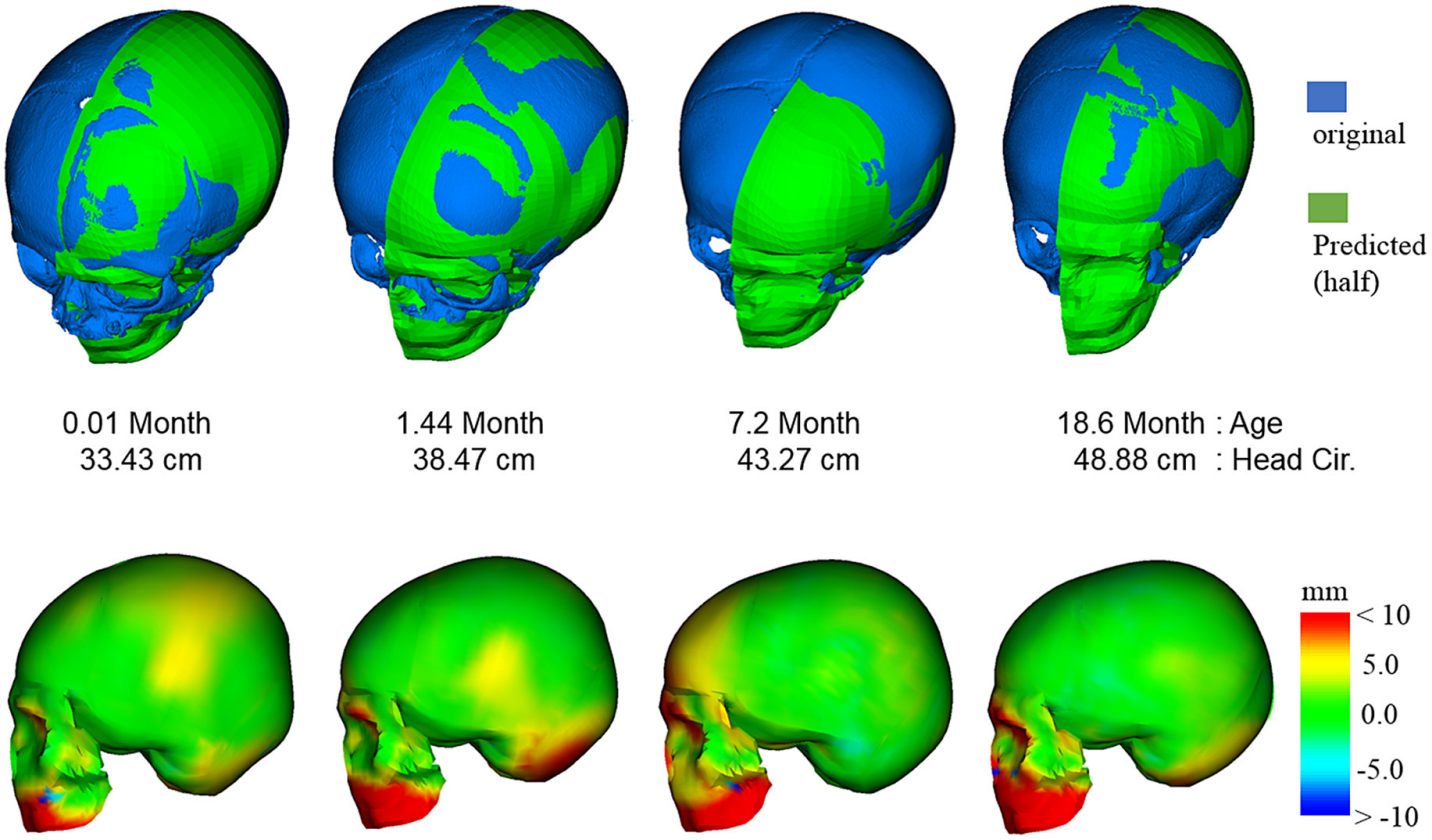
Comparison between model predicted skull geometry and segmented CT geometry.

The landmarks of fifteen subjects, who were not included in the pool of the statistical model, were predicted with given ages and head circumferences using the statistical model presented here. Fig 4 shows signed errors between the predicted landmark coordinates and the actual data collected from CT data of the same subjects. The result showed a good potential in accuracy. Errors in X coordinates had the mean value of 0.07 mm with a standard deviation (SD) of 2.26, Y errors had the mean of 0.13 mm (SD 3.05), and Z errors had 0.93 mm (SD 4.06). Fig 5 shows a comparison between the model-predicted skull geometries and segmented CT geometries from 4 subjects.

### Skull Size and Shape Changes for Children from 0 to 3 YO

**Fig 6:**
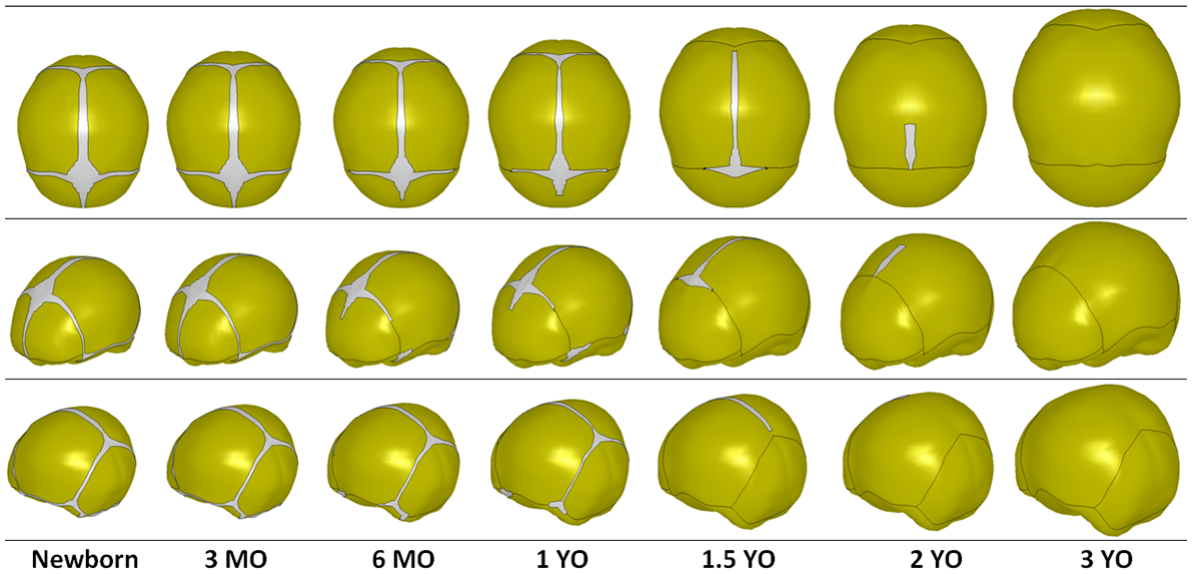
Skull size/shape and suture changes by age.

**Fig 7:**
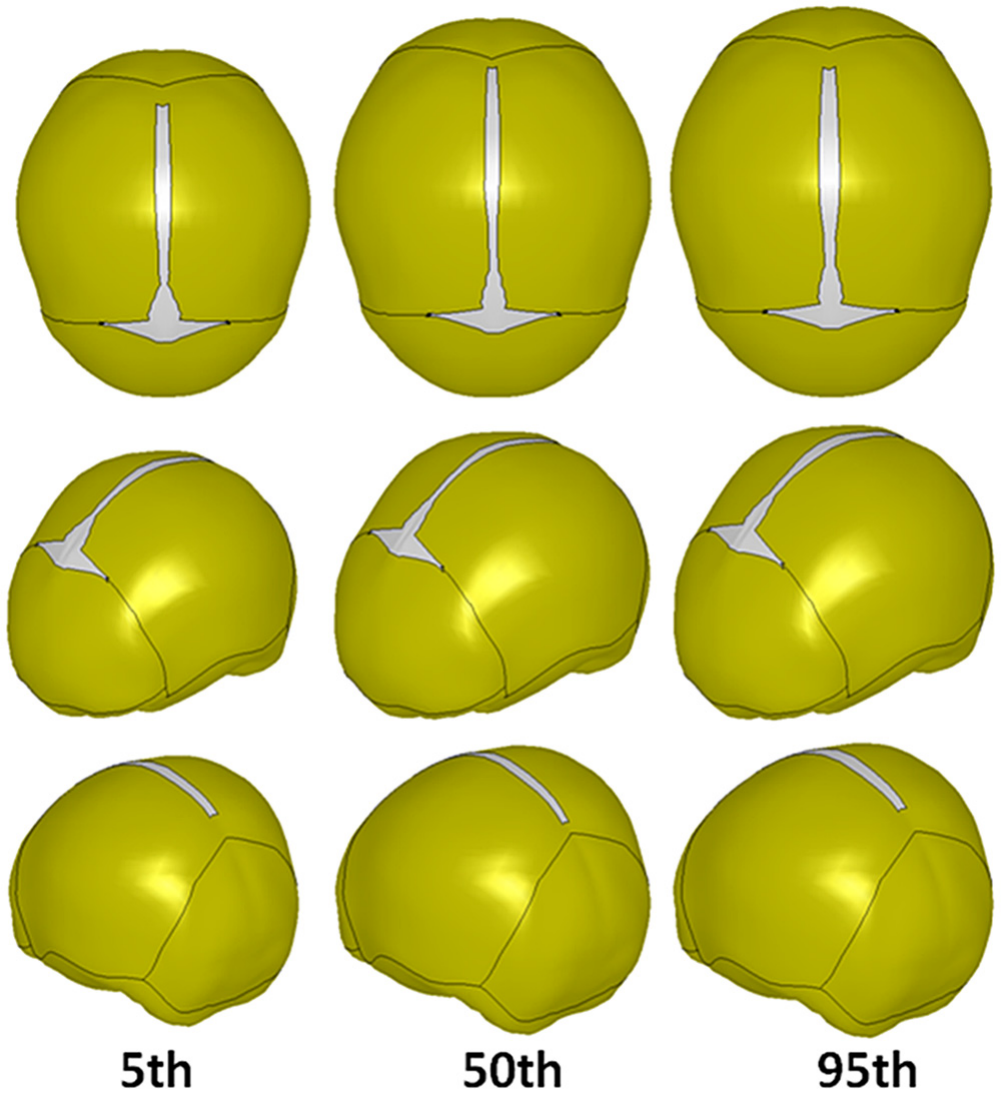
Head size/shape and suture variation among 1.5 YO children.

Seven skull geometries representing 50th percentile skulls at different ages (newborn, 3 MO, 6 MO, 1 YO, 1.5 YO, 2 YO, and 3 YO) were generated using the skull geometry regression model with the mean head circumference (CirOffset = 0). Landmark spatial locations for the 50th percentile skulls of four ages (newborn, 1 YO, 2 YO, and 3 YO) were provided in Table A in the S1 Appendix. To illustrate the changes of the skull size/shape with increasing age, radial basis function was used to generate the skull surfaces based on the landmark locations as shown in Fig 6. It is clear that the skull size increases from newborn to 3 YO, but the growth speed slows down relatively toward 3 YO. To show the head size and shape variation at the same age, the skull geometries for the 1.5 YO children with different head circumferences were also generated using the regression model by setting the age at 1.5 YO and the CirOffset at the values corresponding to the 5th, 50th, 95th percentile head circumferences for 1.5 YO children. The landmark spatial locations associated with these three models are provided in Table B in S1 Appendix, and the corresponding geometry illustrations are shown in Fig 7.

The suture width values for the 50^th^ percentile children at different ages (newborn, 3 MO, 6 MO, 1 YO, 1.5 YO, 2 YO, and 3 YO) at 32 landmarks (Fig 2) are provided in Table C in S1 Appendix, and visual illustrations are shown in Fig 6. Note that if the model-predicted suture width values are negative, they were amended to 0. It is evident that the suture gradually closes from newborn to 3 YO. However, the suture closing speeds are very different across the skull. In particular, the squamosal suture, the inferior region of lambdoid suture and coronal suture, and the region of the sagittal suture near the frontal cranium close more rapidly than other parts of the sutures.

For children from newborn to 3 MO, none of the sutures were closed. For children 4–7 MO, suture closure occurred in the inferior region of the coronal suture, most squamosal suture, as well as in some portions of the sagittal suture near the frontal cranium. The fonticulus sphenoideus and fonticulus mastoideus also closed over this age range. For children from 8 to 12 MO, all the squamosal sutures, a large portion of the coronal suture towards the superior region, the lambdoid suture under the occipital cranium, and the posterior suture closed. For children from 1 to 1.5 YO, suture closure occurred in the whole lambdoid suture, coronal suture, sagittal suture near the occipital cranium and the anterior fontanel. For children from 1.5 to 2 YO, nearly all sutures closed except for a small portion of the sagittal suture behind the anterior fontanel. For children older than 2 YO, all sutures and fontanels are closed.

The suture width variation represented by the 32 landmarks on the suture for children 1.5 YO with 5^th^, 50^th^, 95^th^ percentile head circumferences are provided in Table C in S1 Appendix, and visual illustrations are shown in Fig 7.

The results from the linear mixed model (Table 1) showed that age, head circumference, and landmark locations as well as the interaction between each two of the three parameters are all significant predictors of suture width.

**Table 1:** Results from the linear mixed regression model.

Effect	Suture Width				Skull Thickness			
	Num DF	Den DF	F Value	Pr>F	Num DF	Den DF	F Value	Pr>F
**Landmark**	31	1643	4.80	<.0001	23	1219	2.05	0.0026
**Age**	1	52	31.74	<.0001	1	53	61.69	<.0001
**Circumference**	1	52	68.56	<.0001	1	53	7.37	0.0089
Age*Landmark	31	1643	7.53	<.0001	23	1219	4.76	<.0001
Circumference*Landmark	31	1643	3.03	<.0001	23	1219	2.53	<.0001
Age*Circumference	1	52	29.25	<.0001	-	-	-	-

### Skull Thickness Change for Children from 0 to 3 YO

**Fig 8:**
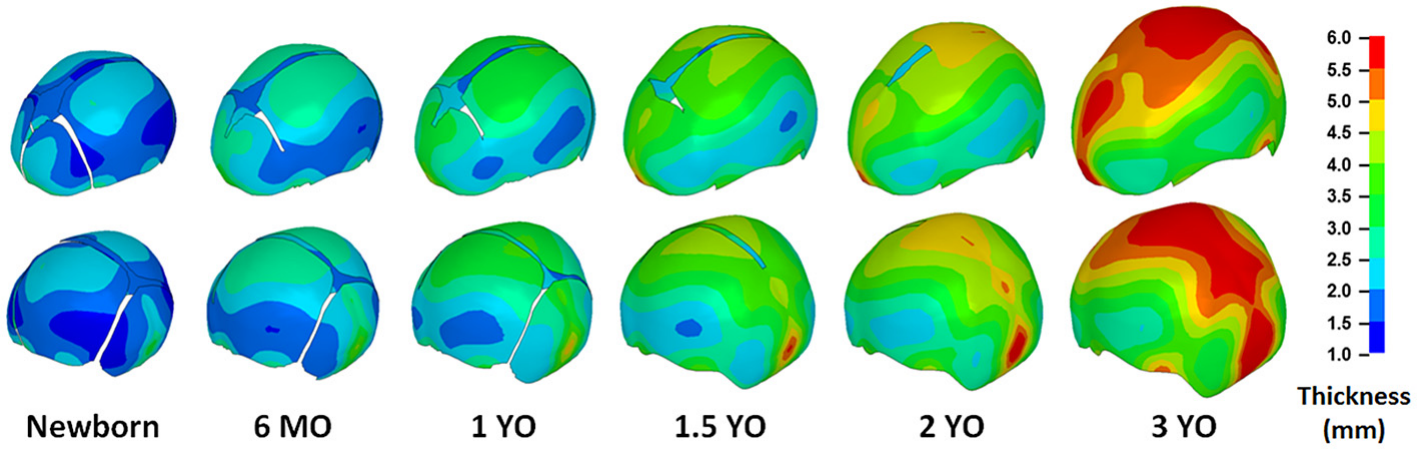
Skull thickness distribution by age. The models shown here were generated by morphing a template mesh into the model-predicted skull geometry using a Radial Basis Function. The color contours were generated based on the skull thickness values associated with each node on the morphed mesh. The quantitative skull thickness data corresponding to each landmark location can be found in Table D in the S1 Appendix.

**Fig 9:**
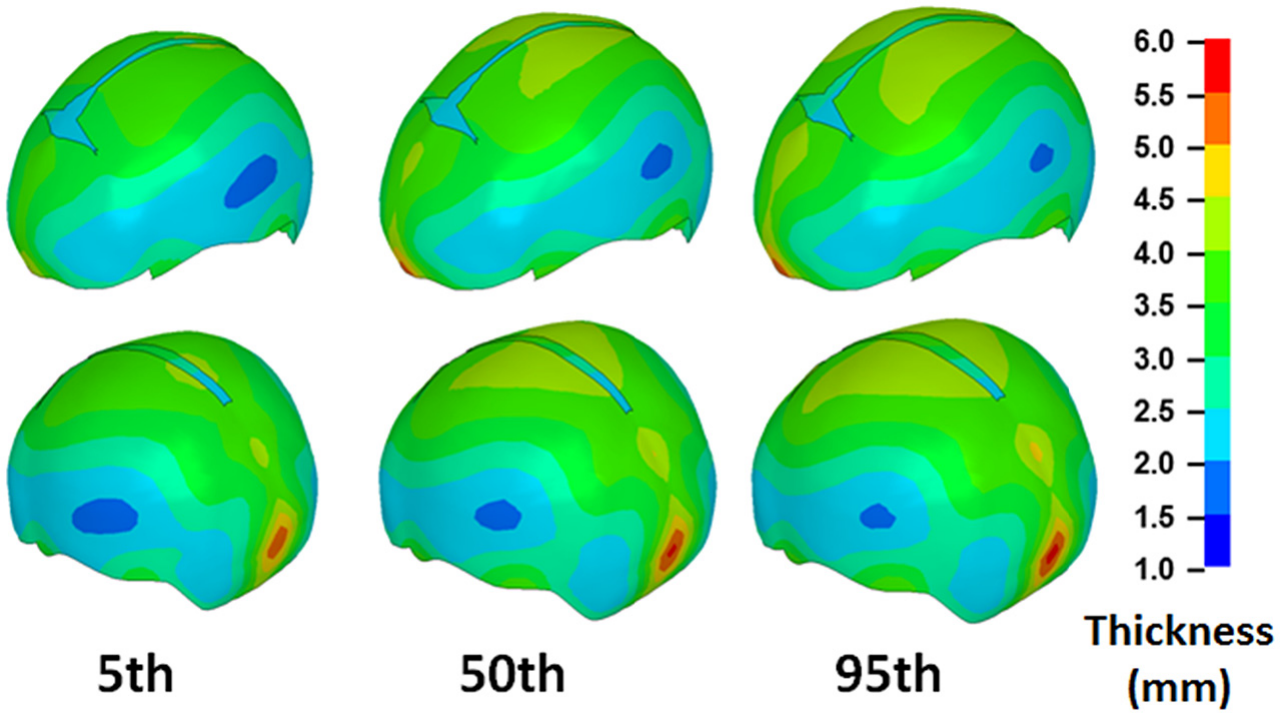
Skull thickness distribution variation among 1.5 YO children with a range of head circumference. The models shown here were generated by morphing a template mesh into the model-predicted skull geometry using a Radial Basis Function. The color contours were generated based on the skull thickness values associated with each node on the morphed mesh. The quantitative skull thickness data corresponding to each landmark location can be found in Table D in the S1 Appendix.

The skull thickness distributions representing 50^th^ percentile children at different ages (newborn, 6 MO, 1 YO, 1.5 YO, 2 YO, and 3 YO) were predicted by the regression model and the skull thickness at the landmark locations are provided in Table D in S1 Appendix. Fig 8 illustrates the skull thickness distributions for children at different ages, in which the skull thickness values at locations other than the landmarks were interpolated based on the thickness values at the landmark locations using radial basis functions [21, 22]. It is shown that the skull thickness increases from newborn to 3 YO, but it is non-uniformly distributed across the skull. In particular, the skull thickness values in the occipital region are much higher than those in the frontal and parietal regions. The skull thickness for 1.5 YO children with 5^th^, 50^th^, 95^th^ percentile head circumferences are shown in Fig 9, and the values are provided in Table D in S1 Appendix.

The results from the linear mixed model (Table 1) showed that age, head circumference, and landmark locations as well as two interaction terms are all significant predictors of skull thickness. The significant effects from landmark location, the interactions between age and landmark location, and the interaction between head circumference and landmark locations indicated that the skull thickness is not uniformly distributed among the skull, and age and head circumference may affect this non-uniform distribution.

## Discussion

Early studies on pediatric head geometry mainly focused on global dimensions, such as the head length, width, height, and circumference [27–29] by measuring a large number of volunteer subjects. Detailed information on 3-dimensional size/shape, suture width, and skull thickness of pediatric heads as well as their variations among children with different ages are largely lacking. In a previous study by Li et al. [21, 22], a statistical skull geometry model for 0–3 MO infants was developed based on head CT data from 11 subjects. In that statistic model, age was considered as the only predictor for skull geometry. The small sample size and small age range limit the application of that study. Moreover, that study was focused on developing a parametric pediatric head FE model, so the morphological details, including head size/shape, skull thickness, and suture width were not presented quantitatively. In order to extend the age ranges with more subjects and better represent the morphological variations for pediatric skulls, in the present study, an improved statistical model of skull geometry was developed based on landmark data extracted from 56 head CT scans that is valid for a larger range of ages (0–3 YO) and that better considers the effects of variations in head size/shape, suture width, and skull thickness. Because landmarks were identified not only on the skull, but also along the skull-suture borders, the model developed in this study can accurately represent suture width and skull thickness distribution. The variations in skull geometry among a specific age and head circumference have also been quantified through statistical analyses.

Another important contribution of this study is the characterization of skull thickness distributions. The skull thicknesses in almost all previous pediatric head FE models were assumed to be constant. For example, the skull thickness values of the newborn head in studies by Coats et al. [13] and Roth et al. [16] were assigned as 1.2 and 1.4 mm, respectively. These values are slightly lower than the average value reported in the current study. The skull thickness of the 3 YO head model in a study by Roth et al. [17] was 4 mm, which is fairly consistent with the average skull thickness of the 3 YO children in the current study. However, considering the relative large variation found in the skull thickness at different locations, a single value is probably not enough to describe the skull thickness distribution in 0–3 YO children, and a uniform change in skull thickness cannot represent the skull thickness variation either. Therefore, whether a constant skull thickness is acceptable for accurately predicting the head injury risk needs further investigation. The skull thickness distribution reported in the current study provided more accurate skull thickness representations, which can be applied in future pediatric head FE models. In a previous study by McPherson & Kriewall [30], it was reported that the center of the parietal bone is slightly thicker than the periphery area based on the measurement of a real fetus. This finding is contradictory to the result of the current study in which the center of the parietal bone seems slightly thinner than the regions near the sutures. This is likely because (1) McPherson & Kriewall [30] only investigated a single subject; (2) the subject is a fetus with very thin skull and the difference between the center and the periphery area of the parietal bone was within 0.2 mm in the study of McPherson & Kriewall [30]. In our current study, the skull thickness difference within the parietal bone is also very small for the newborn. However, with increase in age, the increase in the thickness at the center of the parietal bone is slower than that at the periphery area.

In the literature, a few studies have analyzed medical image data to establish statistical pediatric head geometry models at different ages. Danelson et al. [31] conducted a statistical analysis on brain shape and size using MR scans from subjects ranging from newborn to adult and established a regression model to predict brain geometry changes with age. The skull geometry was not quantified in that study. Loyd et al. [6] quantitatively illustrated the relationships between pediatric head global dimension changes (e.g. head length, width, and circumference) and age. That study focused on producing pediatric head contours for children with different ages and compared them with the current ATDs. The pediatric head contours at each age group were obtained by averaging the head contours under a similar age group. Both Danelson et al [31] and Loyd et al [6] stressed the importance of considering the pediatric head shape variations when developing ATDs and FE models, but suture and skull thickness were not considered in either of their geometry models.

The statistical skull geometry model presented here is broadly applicable and can be readily extended to a wide range of applications. One of the convincing applications is to be used in finite element (FE) analyses. We expect that the model will be useful for rapidly generating patient-specific FE models by morphing a template FE skull model based on the predicted landmarks and geometry information with only a few predictors such as age and head circumference. In addition, since the model provides a parametric way to control the geometry, the FE results can yield reliable injury risk curves with respect to each predictor's variation. These curves can be used to simulate and predict patterns of injuries on a skull in specific accidental conditions, such as car crashes and child-falling cases.

This study has several limitations. First, to collect the valid data from the CTs of the pediatric heads, the data were screened and controlled as much as possible to not include children with disorders that could affect their growth. However, it is important to note that these children are not completely healthy children. Second, the skull thickness was manually measured in the skull geometry reconstructed from the CT scans. Although the CT images sustained a resolution of 0.273 to 0.410 mm/pixel, there are 1.25 mm or 2.5 mm intervals between CT slices. As a result, the skull thickness measured on the sides of the skull should be fairly accurate, while the accuracy may be lower on the top of the head where the thickness are often measured through the CT intervals. Third, the landmarks were manually selected based on the anatomical features on the suture and evenly distributed on the skull. Errors during this manual process would tend to smooth these features in the resulting model. Due to the limited number of landmarks, more detailed fractal dimension of the suture could not be quantified. An improvement on the current methods would be to automatically or semi-automatically locate and extract landmarks from the pediatric head CT scans, but any automated method would need to be carefully validated against expert human judgment. Finally, the analysis is limited by the sample size, thus other subject covariates, such as gender, cannot be considered in this study. More CT scans are needed to quantify the other effects on pediatric skull geometry. Nonetheless, the current study provided a valuable method to quantify the 3-dimensional pediatric skull geometry that can be directly used for future modeling purpose. It should also be mentioned that, in this study, we only focused on the statistical geometry of the skull, and the material properties (e.g. density, Young’s modulus, etc) of the skull and other components were not considered and will be investigated in our future studies.

## Concluding Remarks

In this study, image analysis and statistical analysis were conducted to develop a statistical three-dimensional skull geometry model for 0–3 YO children based head CT scans from 56 child subjects. This model can predict skull geometrical features, such as head size/shape, skull thickness, and suture width by age and head circumference. The statistical model developed in this study can be used as the geometric basis for developing parametric pediatric head FE models and child ATDs.

## Supporting Information

S1 Appendix Principal Component Analysis (PCA) method and Tables A-D. (DOCX)
