# Supplementary material for: A Statistical Skull Geometry Model for Children 0-3 Years Old
Source: PLoS One. 2015 May 18;10(5):e0127322. doi: 10.1371/journal.pone.0127322 (PMC4436309; doi:10.1371/journal.pone.0127322)
Supplement: S1 Appendix — (DOCX) [file pone.0127322.s001.docx]

# APPENDIX

**Principal Component Analysis (PCA) method**

Let ***G*** and ***g*** be the geometry matrix and an individual vector with the length of *l* respectively. The geometric matrix ***G*** is first centered by subtracting the mean from each of the individual’s ***g_i_***. *G* can be decomposed as follows,

$G=SP$ (1)

$S=GP^{T}$ (2)

where ***S*** is an *N × l* matrix called principal component (PC) scores, and ***P*** is a matrix composed by the eigenvectors of ***G***, which is an *l × l* normalized matrix. *T* indicates a transpose of a matrix.

The matrix ***G*** can be closely approximated by taking the first k principal components,

$G^{*}=S_{\mathbf{k}}P^{\mathbf{T}}_{\mathbf{k}}$ (3)

where ***G*^*^** is the approximation of ***G***, ***S*_k_** is the first k columns of ***S***, and ***P*_k_** is the first k columns of **P**.

Any individual’s coordinates of landmarks can be obtained based on the equation as,

$g_{i}^{*}=\bar{g}+S_{iK}P_{K}^{T}$ (4)

where $\mathbf{S}_{iK}$ is the row of matrix $\mathbf{S}_{N}$corresponding to the *i*^th^ individual’s principal component scores.

Table A. Landmark spatial location for the 50^th^ percentile skulls at different ages (Unit: mm)

| **Age** | **0MO** | | | **1YO** | | | **2YO** | | | | **3YO** | | |
| --- | --- | --- | --- | --- | --- | --- | --- | --- | --- | --- | --- | --- | --- |
| **Landmark no.** | X | Y | Z | X | Y | Z | X | Y | Z | X | | Y | Z |
| **B01** | 30.77 | 8.46 | 0.63 | 31.9 | 10.61 | 0.58 | 33.02 | 12.75 | 0.54 | 34.15 | | 14.89 | 0.49 |
| **B02** | 18.35 | -1.36 | 14.55 | 18.2 | -0.5 | 16.56 | 18.05 | 0.35 | 18.57 | 17.9 | | 1.21 | 20.58 |
| **B03** | 28.31 | 9.74 | 34.41 | 28.91 | 10.93 | 37.85 | 29.51 | 12.13 | 41.3 | 30.11 | | 13.33 | 44.74 |
| **B04** | 17.92 | 11.62 | 45.33 | 16.92 | 13.84 | 51.81 | 15.91 | 16.06 | 58.29 | 14.9 | | 18.28 | 64.76 |
| **B05** | 43.11 | 21.58 | 11.58 | 45.34 | 24.48 | 13.06 | 47.57 | 27.37 | 14.54 | 49.8 | | 30.27 | 16.02 |
| **B06** | 54.19 | 53.49 | 27.34 | 56.95 | 57.59 | 32.7 | 59.7 | 61.7 | 38.06 | 62.45 | | 65.8 | 43.42 |
| **B07** | 50.78 | 49.15 | 46.78 | 52.56 | 52.92 | 52.54 | 54.34 | 56.69 | 58.3 | 56.12 | | 60.46 | 64.06 |
| **B08** | 39.99 | 45.07 | 59.62 | 40.69 | 49.02 | 66.32 | 41.39 | 52.97 | 73.02 | 42.1 | | 56.92 | 79.73 |
| **B09** | 24.27 | 40.92 | 67.85 | 23.05 | 45.86 | 75.69 | 21.83 | 50.8 | 83.52 | 20.61 | | 55.74 | 91.35 |
| **B10** | 35.83 | 58.26 | 69.52 | 36.31 | 63.05 | 77.25 | 36.79 | 67.83 | 84.98 | 37.27 | | 72.62 | 92.72 |
| **B11** | 22.9 | 68.82 | 77.78 | 23.73 | 76.71 | 86.24 | 24.57 | 84.59 | 94.71 | 25.4 | | 92.48 | 103.17 |
| **B12** | 52.78 | 75.47 | 49.55 | 56.43 | 81.69 | 55.64 | 60.08 | 87.91 | 61.74 | 63.73 | | 94.13 | 67.84 |
| **B13** | 49.07 | 93.66 | 32.59 | 52.73 | 103.36 | 37.39 | 56.38 | 113.05 | 42.19 | 60.04 | | 122.75 | 46.98 |
| **B14** | 42.27 | 100.59 | 47.59 | 45.19 | 110.41 | 54.02 | 48.11 | 120.24 | 60.44 | 51.03 | | 130.06 | 66.86 |
| **B15** | 32.16 | 101.93 | 59.84 | 34.3 | 111.83 | 67.51 | 36.43 | 121.72 | 75.19 | 38.57 | | 131.62 | 82.87 |
| **B16** | 18.85 | 100.48 | 69.16 | 19.68 | 110.19 | 77.38 | 20.52 | 119.9 | 85.61 | 21.35 | | 129.62 | 93.83 |
| **B17** | 12.8 | 114.63 | -3.29 | 15 | 128.13 | -3.64 | 17.19 | 141.63 | -3.98 | 19.39 | | 155.13 | -4.33 |
| **B18** | 24.96 | 120.85 | 20.8 | 27.53 | 133.52 | 25.68 | 30.1 | 146.18 | 30.56 | 32.66 | | 158.84 | 35.43 |
| **B19** | 10.18 | 123.41 | 41.21 | 10.25 | 135.73 | 48.58 | 10.32 | 148.05 | 55.94 | 10.39 | | 160.37 | 63.3 |
| **B20** | -0.68 | 122.72 | 43.69 | -1.75 | 134.46 | 51.5 | -2.82 | 146.21 | 59.31 | -3.9 | | 157.95 | 67.12 |
| **B21** | -0.7 | 126.78 | 31.35 | -1.6 | 139.73 | 37.52 | -2.5 | 152.68 | 43.68 | -3.4 | | 165.63 | 49.84 |
| **B22** | -0.56 | 127.01 | 18.54 | -1.45 | 140.36 | 22.7 | -2.33 | 153.71 | 26.86 | -3.22 | | 167.06 | 31.02 |
| **B23** | -0.3 | 121.61 | 7.13 | -1.21 | 135.29 | 8.97 | -2.12 | 148.96 | 10.81 | -3.03 | | 162.63 | 12.65 |
| **B24** | -0.23 | 114.96 | -3.54 | -1.04 | 128.18 | -3.63 | -1.85 | 141.41 | -3.72 | -2.66 | | 154.63 | -3.81 |
| **C01** | 43.16 | 33.6 | -0.17 | 44.66 | 38.24 | 0.56 | 46.16 | 42.88 | 1.29 | 47.66 | | 47.52 | 2.03 |
| **C02** | -0.11 | 21.17 | 59.4 | -0.8 | 25.51 | 67.35 | -1.49 | 29.86 | 75.29 | -2.17 | | 34.21 | 83.24 |
| **C03** | -0.66 | 113.4 | 59.03 | -1.83 | 125.11 | 66.06 | -2.99 | 136.81 | 73.08 | -4.15 | | 148.52 | 80.11 |
| **C04** | 40.87 | 100.02 | 3.8 | 45.22 | 111.54 | 6.94 | 49.58 | 123.06 | 10.07 | 53.94 | | 134.58 | 13.21 |
| **S01** | 0 | 0 | 0 | 0 | 0 | 0 | 0 | 0 | 0 | 0 | | 0 | 0 |
| **S02** | 0.09 | -3.63 | 14.3 | -0.15 | -3.37 | 16.12 | -0.39 | -3.11 | 17.94 | -0.63 | | -2.85 | 19.76 |
| **S03** | 0.01 | -1.95 | 26.23 | -0.29 | -1.53 | 29.6 | -0.58 | -1.11 | 32.98 | -0.88 | | -0.68 | 36.35 |
| **S04** | -0.05 | 2.84 | 37.39 | -0.44 | 3.85 | 42.37 | -0.83 | 4.87 | 47.36 | -1.23 | | 5.88 | 52.35 |
| **S05** | -0.04 | 9.37 | 47.2 | -0.54 | 11.34 | 53.62 | -1.04 | 13.3 | 60.04 | -1.54 | | 15.27 | 66.46 |
| **S06** | 0 | 16.43 | 55.27 | -0.68 | 20.06 | 62.97 | -1.36 | 23.7 | 70.66 | -2.04 | | 27.34 | 78.36 |
| **S07** | -0.1 | 25.19 | 62.46 | -0.73 | 29.85 | 70.44 | -1.37 | 34.52 | 78.42 | -2 | | 39.18 | 86.4 |
| **S08** | -0.16 | 37.05 | 71.28 | -1 | 43.48 | 79.79 | -1.83 | 49.92 | 88.3 | -2.67 | | 56.35 | 96.81 |
| **S09** | -0.22 | 52.66 | 78.73 | -1.31 | 60.76 | 87.46 | -2.41 | 68.87 | 96.18 | -3.5 | | 76.97 | 104.91 |
| **S10** | -0.44 | 73.7 | 81.45 | -1.41 | 84.18 | 89.8 | -2.38 | 94.67 | 98.16 | -3.35 | | 105.15 | 106.51 |
| **S11** | -0.61 | 96 | 75.07 | -1.67 | 106.72 | 82.65 | -2.73 | 117.44 | 90.23 | -3.79 | | 128.15 | 97.8 |
| **S12** | -0.76 | 107.19 | 67.02 | -1.78 | 117.74 | 74.4 | -2.81 | 128.29 | 81.78 | -3.83 | | 138.84 | 89.16 |
| **S13** | 13.8 | 117.14 | 52.44 | 13.73 | 128.97 | 59.46 | 13.66 | 140.79 | 66.49 | 13.59 | | 152.61 | 73.51 |
| **S14** | 25.02 | 117.31 | 44.36 | 26.35 | 129.14 | 51.05 | 27.68 | 140.98 | 57.74 | 29 | | 152.81 | 64.44 |
| **S15** | 31.45 | 115.97 | 35.64 | 33.43 | 128.22 | 40.81 | 35.41 | 140.47 | 45.98 | 37.39 | | 152.73 | 51.15 |
| **S16** | 36.5 | 112.97 | 26.38 | 38.85 | 125.1 | 30.97 | 41.21 | 137.23 | 35.56 | 43.56 | | 149.36 | 40.15 |
| **S17** | 39.54 | 108.12 | 17.67 | 42.07 | 120.12 | 21.19 | 44.59 | 132.13 | 24.71 | 47.11 | | 144.13 | 28.23 |
| **S18** | 40.18 | 102.87 | 8.73 | 43.83 | 114.95 | 11.73 | 47.48 | 127.04 | 14.73 | 51.13 | | 139.12 | 17.73 |
| **S19** | 44.8 | 93.58 | 3.83 | 49.66 | 103.68 | 7.23 | 54.53 | 113.79 | 10.63 | 59.39 | | 123.89 | 14.03 |
| **S20** | 48.13 | 81.97 | 2.46 | 53.45 | 90.51 | 6.1 | 58.78 | 99.05 | 9.75 | 64.1 | | 107.59 | 13.4 |
| **S21** | 52.63 | 69.61 | 6.32 | 57.34 | 76.44 | 10.93 | 62.06 | 83.27 | 15.55 | 66.77 | | 90.1 | 20.16 |
| **S22** | 52.19 | 56.2 | 6.33 | 55.87 | 61.44 | 10.91 | 59.54 | 66.68 | 15.48 | 63.22 | | 71.92 | 20.06 |
| **S23** | 45.21 | 33.01 | 5.76 | 47.5 | 36.84 | 7.44 | 49.8 | 40.67 | 9.12 | 52.09 | | 44.49 | 10.79 |
| **S24** | 47.91 | 31.59 | 17.27 | 50.14 | 35.29 | 19.94 | 52.37 | 38.99 | 22.6 | 54.6 | | 42.69 | 25.27 |
| **S25** | 47.38 | 29.11 | 29.25 | 49.47 | 32.47 | 33.19 | 51.55 | 35.84 | 37.13 | 53.64 | | 39.21 | 41.07 |
| **S26** | 42.98 | 27.17 | 40.43 | 44.39 | 30.28 | 45.38 | 45.79 | 33.38 | 50.33 | 47.19 | | 36.48 | 55.28 |
| **S27** | 34.75 | 26.17 | 50.4 | 35.07 | 28.83 | 56.1 | 35.39 | 31.48 | 61.8 | 35.71 | | 34.14 | 67.5 |
| **S28** | 23.16 | 25.5 | 57.03 | 22.28 | 28.13 | 63.58 | 21.4 | 30.75 | 70.13 | 20.52 | | 33.38 | 76.69 |
| **S29** | 30.19 | 95.69 | -13.99 | 33.9 | 106.96 | -15.09 | 37.62 | 118.23 | -16.2 | 41.33 | | 129.5 | -17.3 |
| **S30** | 24.53 | 103.47 | -12.23 | 27.77 | 115.76 | -13.52 | 31.01 | 128.05 | -14.8 | 34.24 | | 140.34 | -16.09 |
| **S31** | 13.16 | 106.14 | -13.66 | 14.5 | 118.94 | -14.68 | 15.85 | 131.73 | -15.7 | 17.19 | | 144.53 | -16.72 |
| **S32** | -0.03 | 106.14 | -14.23 | -0.81 | 118.08 | -15.31 | -1.6 | 130.01 | -16.38 | -2.38 | | 141.94 | -17.45 |

Table B. Landmark spatial location for skulls of 1.5YO children at different sizes (Unit: mm)

| **Percentile** | **5^th^ percentile** | | | **50^th^ percentile** | | | **95^th^ percentile** | | |
| --- | --- | --- | --- | --- | --- | --- | --- | --- | --- |
| **Landmark no.** | X | Y | Z | X | Y | Z | X | Y | Z |
| **B01** | 30.77 | 11.12 | 0.43 | 32.46 | 11.68 | 0.56 | 34.16 | 12.24 | 0.69 |
| **B02** | 17.08 | -0.25 | 16.39 | 18.13 | -0.07 | 17.57 | 19.18 | 0.1 | 18.76 |
| **B03** | 27.88 | 10.12 | 37.42 | 29.21 | 11.53 | 39.57 | 30.55 | 12.96 | 41.75 |
| **B04** | 16.58 | 13.26 | 52.33 | 16.41 | 14.95 | 55.05 | 16.25 | 16.66 | 57.79 |
| **B05** | 43.89 | 24.41 | 12.74 | 46.46 | 25.92 | 13.8 | 49.05 | 27.46 | 14.87 |
| **B06** | 55.53 | 55.86 | 33.75 | 58.32 | 59.65 | 35.38 | 61.15 | 63.47 | 37.02 |
| **B07** | 51.24 | 51.38 | 52.63 | 53.45 | 54.81 | 55.42 | 55.67 | 58.27 | 58.24 |
| **B08** | 39.46 | 47.63 | 66.61 | 41.04 | 50.99 | 69.67 | 42.64 | 54.38 | 72.76 |
| **B09** | 21.49 | 44.98 | 76.41 | 22.44 | 48.33 | 79.6 | 23.39 | 51.71 | 82.83 |
| **B10** | 34.84 | 61.21 | 77.52 | 36.55 | 65.44 | 81.12 | 38.27 | 69.71 | 84.75 |
| **B11** | 23.01 | 75.02 | 86.43 | 24.15 | 80.65 | 90.48 | 25.3 | 86.33 | 94.56 |
| **B12** | 55.65 | 79.84 | 56.09 | 58.26 | 84.8 | 58.69 | 60.89 | 89.81 | 61.32 |
| **B13** | 51.74 | 102.04 | 38.11 | 54.55 | 108.21 | 39.79 | 57.4 | 114.43 | 41.48 |
| **B14** | 44.27 | 108.62 | 54.56 | 46.65 | 115.32 | 57.23 | 49.05 | 122.09 | 59.92 |
| **B15** | 33.76 | 109.77 | 68.25 | 35.37 | 116.78 | 71.35 | 36.99 | 123.85 | 74.48 |
| **B16** | 19.28 | 107.86 | 78.22 | 20.1 | 115.05 | 81.49 | 20.92 | 122.3 | 84.8 |
| **B17** | 15.66 | 127.38 | -3.39 | 16.1 | 134.88 | -3.81 | 16.54 | 142.45 | -4.23 |
| **B18** | 27.22 | 131.7 | 26.79 | 28.81 | 139.85 | 28.12 | 30.43 | 148.07 | 29.46 |
| **B19** | 9.86 | 133.61 | 49.11 | 10.28 | 141.89 | 52.26 | 10.7 | 150.24 | 55.43 |
| **B20** | -2.07 | 132.02 | 52.39 | -2.29 | 140.34 | 55.4 | -2.5 | 148.73 | 58.45 |
| **B21** | -1.79 | 137.74 | 38.11 | -2.05 | 146.2 | 40.6 | -2.31 | 154.74 | 43.1 |
| **B22** | -1.53 | 138.37 | 23.26 | -1.89 | 147.03 | 24.78 | -2.25 | 155.78 | 26.32 |
| **B23** | -1.33 | 133.84 | 9.39 | -1.67 | 142.12 | 9.89 | -2.01 | 150.48 | 10.39 |
| **B24** | -1.13 | 127.51 | -3.56 | -1.45 | 134.8 | -3.67 | -1.77 | 142.15 | -3.78 |
| **C01** | 43.32 | 38.51 | 1.2 | 45.41 | 40.56 | 0.93 | 47.52 | 42.64 | 0.65 |
| **C02** | -1.22 | 24.47 | 68.09 | -1.14 | 27.69 | 71.32 | -1.07 | 30.93 | 74.59 |
| **C03** | -2.16 | 123.31 | 66.34 | -2.41 | 130.96 | 69.57 | -2.66 | 138.68 | 72.84 |
| **C04** | 44.82 | 111.16 | 8.34 | 47.4 | 117.3 | 8.51 | 50 | 123.5 | 8.67 |
| **S01** | 0 | 0 | 0 | 0 | 0 | 0 | 0 | 0 | 0 |
| **S02** | -0.34 | -3.22 | 15.95 | -0.27 | -3.24 | 17.03 | -0.2 | -3.26 | 18.12 |
| **S03** | -0.45 | -1.7 | 29.63 | -0.43 | -1.32 | 31.29 | -0.41 | -0.93 | 32.96 |
| **S04** | -0.66 | 3.2 | 42.49 | -0.64 | 4.36 | 44.87 | -0.61 | 5.53 | 47.27 |
| **S05** | -0.79 | 10.47 | 54.09 | -0.79 | 12.32 | 56.83 | -0.78 | 14.19 | 59.6 |
| **S06** | -1.11 | 19.2 | 63.66 | -1.02 | 21.88 | 66.82 | -0.92 | 24.6 | 70 |
| **S07** | -1.05 | 28.79 | 71.14 | -1.05 | 32.19 | 74.43 | -1.05 | 35.61 | 77.75 |
| **S08** | -1.46 | 42.76 | 80.54 | -1.41 | 46.7 | 84.04 | -1.37 | 50.67 | 87.57 |
| **S09** | -1.82 | 59.89 | 87.91 | -1.86 | 64.81 | 91.82 | -1.91 | 69.78 | 95.76 |
| **S10** | -1.89 | 83.2 | 90.06 | -1.89 | 89.43 | 93.98 | -1.9 | 95.71 | 97.94 |
| **S11** | -2.17 | 104.88 | 83.19 | -2.2 | 112.08 | 86.44 | -2.23 | 119.35 | 89.72 |
| **S12** | -2.07 | 115.59 | 74.95 | -2.29 | 123.02 | 78.09 | -2.52 | 130.51 | 81.25 |
| **S13** | 13.07 | 126.91 | 59.82 | 13.69 | 134.88 | 62.98 | 14.32 | 142.92 | 66.16 |
| **S14** | 25.62 | 127.25 | 51.75 | 27.01 | 135.06 | 54.4 | 28.42 | 142.94 | 57.08 |
| **S15** | 32.5 | 126.7 | 41.08 | 34.42 | 134.35 | 43.4 | 36.36 | 142.07 | 45.73 |
| **S16** | 37.71 | 123.58 | 31.84 | 40.03 | 131.16 | 33.26 | 42.37 | 138.81 | 34.7 |
| **S17** | 40.69 | 119 | 22.26 | 43.33 | 126.12 | 22.95 | 45.99 | 133.31 | 23.64 |
| **S18** | 43.02 | 114.52 | 12.78 | 45.65 | 120.99 | 13.23 | 48.31 | 127.53 | 13.69 |
| **S19** | 49.47 | 102.93 | 8.84 | 52.09 | 108.73 | 8.93 | 54.74 | 114.6 | 9.01 |
| **S20** | 53.4 | 89.46 | 7.75 | 56.12 | 94.78 | 7.93 | 58.86 | 100.15 | 8.11 |
| **S21** | 56.71 | 75.04 | 12.46 | 59.7 | 79.86 | 13.24 | 62.71 | 84.71 | 14.03 |
| **S22** | 54.84 | 60.38 | 12.88 | 57.7 | 64.06 | 13.2 | 60.59 | 67.77 | 13.51 |
| **S23** | 46.22 | 36.61 | 8.19 | 48.65 | 38.75 | 8.28 | 51.11 | 40.92 | 8.37 |
| **S24** | 48.48 | 34.93 | 20.36 | 51.25 | 37.14 | 21.27 | 54.06 | 39.38 | 22.19 |
| **S25** | 47.96 | 32.04 | 33.38 | 50.51 | 34.16 | 35.16 | 53.08 | 36.3 | 36.95 |
| **S26** | 43.2 | 29.83 | 45.66 | 45.09 | 31.83 | 47.85 | 47 | 33.84 | 50.07 |
| **S27** | 34.2 | 27.98 | 56.09 | 35.23 | 30.15 | 58.95 | 36.27 | 32.35 | 61.84 |
| **S28** | 21.54 | 27.03 | 63.92 | 21.84 | 29.44 | 66.86 | 22.14 | 31.87 | 69.82 |
| **S29** | 34.47 | 106.87 | -14.78 | 35.76 | 112.59 | -15.64 | 37.06 | 118.37 | -16.52 |
| **S30** | 28.22 | 115.79 | -13.12 | 29.39 | 121.9 | -14.16 | 30.57 | 128.07 | -15.21 |
| **S31** | 14.71 | 119.12 | -14.23 | 15.17 | 125.33 | -15.19 | 15.64 | 131.61 | -16.16 |
| **S32** | -0.82 | 118.23 | -15 | -1.2 | 124.04 | -15.84 | -1.59 | 129.91 | -16.7 |

Table C. Suture width values at landmarks for children with different ages and head circumferences

(Unit: mm)

| **Landmark no.** | **0M** | **3MO** | **6MO** | **1YO** | **1.5YO (5th)** | **1.5YO** | **1.5YO (95th)** | **2YO** | **3YO** |
| --- | --- | --- | --- | --- | --- | --- | --- | --- | --- |
| **S01** | 1 | 0.9 | 0 | 0 | 0 | 0 | 0 | 0 | 0 |
| **S02** | 1.1 | 1 | 0 | 0 | 0 | 0 | 0 | 0 | 0 |
| **S03** | 1.3 | 1.1 | 0 | 0 | 0 | 0 | 0 | 0 | 0 |
| **S04** | 2.1 | 1.8 | 0 | 0 | 0 | 0 | 0 | 0 | 0 |
| **S05** | 3.7 | 3.2 | 2.8 | 1.9 | 0 | 0 | 0 | 0 | 0 |
| **S06** | 9.2 | 8.2 | 7.2 | 5.1 | 3.5 | 3.1 | 2.7 | 1.1 | 0 |
| **S07** | 13.6 | 12.1 | 10.7 | 7.8 | 6.1 | 4.9 | 3.6 | 1.9 | 0 |
| **S08** | 4.7 | 4.2 | 3.6 | 2.6 | 1.6 | 1.5 | 1.5 | 0.5 | 0 |
| **S09** | 4.1 | 3.6 | 3.2 | 2.3 | 1.5 | 1.5 | 1.5 | 0.6 | 0 |
| **S10** | 3.9 | 3.5 | 3.1 | 2.2 | 1.5 | 1.4 | 1.3 | 0 | 0 |
| **S11** | 3.6 | 3.2 | 2.9 | 2.1 | 1.4 | 1.3 | 1.2 | 0 | 0 |
| **S12** | 3.6 | 3.2 | 2.8 | 2 | 0 | 0 | 0 | 0 | 0 |
| **S13** | 4.5 | 4 | 3.4 | 2.3 | 0 | 0 | 0 | 0 | 0 |
| **S14** | 3.8 | 3.3 | 2.9 | 1.9 | 0 | 0 | 0 | 0 | 0 |
| **S15** | 3.7 | 3.2 | 2.8 | 1.8 | 0 | 0 | 0 | 0 | 0 |
| **S16** | 3.8 | 3.3 | 2.8 | 1.9 | 0 | 0 | 0 | 0 | 0 |
| **S17** | 3.7 | 3.3 | 2.8 | 1.9 | 0 | 0 | 0 | 0 | 0 |
| **S18** | 4.4 | 3.8 | 3.3 | 2.2 | 0 | 0 | 0 | 0 | 0 |
| **S19** | 2.8 | 2.4 | 2 | 0 | 0 | 0 | 0 | 0 | 0 |
| **S20** | 2.6 | 2.3 | 0 | 0 | 0 | 0 | 0 | 0 | 0 |
| **S21** | 2.5 | 2.1 | 0 | 0 | 0 | 0 | 0 | 0 | 0 |
| **S22** | 2.2 | 1.9 | 0 | 0 | 0 | 0 | 0 | 0 | 0 |
| **S23** | 1.9 | 1.6 | 0 | 0 | 0 | 0 | 0 | 0 | 0 |
| **S24** | 1.8 | 1.6 | 0 | 0 | 0 | 0 | 0 | 0 | 0 |
| **S25** | 2 | 1.7 | 0 | 0 | 0 | 0 | 0 | 0 | 0 |
| **S26** | 3.3 | 2.9 | 2.5 | 0 | 0 | 0 | 0 | 0 | 0 |
| **S27** | 4.4 | 3.9 | 3.4 | 2.3 | 0 | 0 | 0 | 0 | 0 |
| **S28** | 5.6 | 5 | 4.4 | 3.1 | 1.7 | 1.9 | 2.1 | 0 | 0 |
| **S29** | 3.4 | 2.9 | 2.5 | 0 | 0 | 0 | 0 | 0 | 0 |
| **S30** | 1.9 | 1.6 | 0 | 0 | 0 | 0 | 0 | 0 | 0 |
| **S31** | 1.9 | 1.6 | 0 | 0 | 0 | 0 | 0 | 0 | 0 |
| **S32** | 1.8 | 1.6 | 0 | 0 | 0 | 0 | 0 | 0 | 0 |

The suture width values below 0.5 mm were considered as zero in this analysis due to the CT resolution and element size constraints in future FE analysis. Therefore, zero value of suture width in this table does not necessarily mean a complete suture closure.

Table D. Skull thickness values at landmarks for children with different ages and head circumferences

(Unit: mm)

| **Landmark no.** | **0M** | **3MO** | **6MO** | **1YO** | **1.5YO (5th)** | **1.5YO** | **1.5YO (95th)** | **2YO** | **3YO** |
| --- | --- | --- | --- | --- | --- | --- | --- | --- | --- |
| **B01** | 2.8 | 2.9 | 2.9 | 3 | 2.9 | 3.1 | 3.3 | 3.2 | 3.4 |
| **B02** | 2.2 | 2.3 | 2.4 | 2.6 | 2.7 | 2.7 | 2.8 | 2.9 | 3.3 |
| **B03** | 2.2 | 2.3 | 2.5 | 2.7 | 2.8 | 3 | 3.2 | 3.3 | 3.8 |
| **B04** | 2.1 | 2.4 | 2.6 | 3.2 | 3.4 | 3.7 | 3.9 | 4.2 | 5.2 |
| **B05** | 1.8 | 1.9 | 1.9 | 2.1 | 2.2 | 2.3 | 2.3 | 2.4 | 2.7 |
| **B06** | 1.6 | 1.7 | 1.8 | 2 | 2.2 | 2.3 | 2.3 | 2.5 | 2.9 |
| **B07** | 2 | 2.1 | 2.3 | 2.6 | 2.6 | 2.9 | 3.2 | 3.2 | 3.8 |
| **B08** | 2.4 | 2.7 | 2.9 | 3.4 | 3.6 | 3.8 | 4.1 | 4.3 | 5.3 |
| **B09** | 2.5 | 2.7 | 3 | 3.4 | 3.6 | 3.8 | 4.1 | 4.3 | 5.2 |
| **B10** | 2.4 | 2.6 | 2.8 | 3.3 | 3.7 | 3.8 | 4 | 4.3 | 5.3 |
| **B11** | 2.3 | 2.6 | 2.9 | 3.5 | 3.9 | 4.1 | 4.3 | 4.7 | 5.8 |
| **B12** | 1.6 | 1.7 | 1.9 | 2.2 | 2.4 | 2.5 | 2.6 | 2.8 | 3.4 |
| **B13** | 1.2 | 1.3 | 1.4 | 1.7 | 1.8 | 1.9 | 1.9 | 2.1 | 2.5 |
| **B14** | 1.4 | 1.5 | 1.7 | 2 | 2.3 | 2.3 | 2.3 | 2.6 | 3.3 |
| **B15** | 2 | 2.1 | 2.3 | 2.7 | 2.9 | 3 | 3.2 | 3.4 | 4.1 |
| **B16** | 2.3 | 2.5 | 2.7 | 3.2 | 3.5 | 3.7 | 3.9 | 4.1 | 5.1 |
| **B17** | 1.8 | 2 | 2.1 | 2.5 | 2.8 | 2.9 | 2.9 | 3.3 | 4 |
| **B18** | 1.5 | 1.7 | 1.8 | 2 | 2.4 | 2.3 | 2.3 | 2.6 | 3.1 |
| **B19** | 1.7 | 2 | 2.3 | 2.9 | 3.3 | 3.5 | 3.7 | 4.1 | 5.2 |
| **B20** | 2.5 | 2.8 | 3.2 | 3.9 | 4.4 | 4.6 | 4.8 | 5.3 | 6.8 |
| **B21** | 2.3 | 2.6 | 2.8 | 3.3 | 3.7 | 3.8 | 3.9 | 4.3 | 5.3 |
| **B22** | 2.6 | 3 | 3.4 | 4.1 | 5 | 4.9 | 4.8 | 5.6 | 7.1 |
| **B23** | 3.6 | 4 | 4.4 | 5.2 | 5.8 | 6 | 6.3 | 6.8 | 8.4 |
| **B24** | 3.1 | 3.4 | 3.7 | 4.3 | 4.8 | 5 | 5.1 | 5.6 | 6.8 |
